# 1274. Long-term Sustainability of Improvements in Antibiotic Prescribing for Inpatient Skin and Soft Tissue Infections after Implementation of Local Treatment Guidelines

**DOI:** 10.1093/ofid/ofad500.1114

**Published:** 2023-11-27

**Authors:** Kira Voyer, Katherine C Shihadeh, Margaret Cooper, Timothy C Jenkins

**Affiliations:** Children's Hospital Colorado, Denver, Colorado; Denver Health, Denver, Colorado; Denver Health Medical Center, Denver, Colorado; Denver Health and Hospital Authority, Denver, Colorado

## Abstract

**Background:**

Institutional guidelines for the management of inpatient skin and soft tissue infections (SSTIs) were implemented at an academic public safety-net hospital in 2009. This led to an immediate and marked decrease in unnecessary use of broad-spectrum antibiotics and durations of therapy. The objective of this study was to determine if these changes in prescribing have been sustained over time.

**Methods:**

This was a retrospective review of patients hospitalized with cellulitis or abscess at Denver Health Medical Center between July 2016 and August 2022 (maintenance period). Case identification and data collection methods were identical to those used in a previous publication describing pre-intervention (January to December 2007) and intervention (July 2009 to July 2010) period data. The main outcomes were the proportion of patients exposed to an antibiotic with broad-spectrum gram-negative or antipseudomonal activity and the median duration of therapy.

**Results:**

A total of 186 patients from the maintenance period were included for comparison with those from the pre-intervention (n = 169) and intervention (n = 175) periods. In the maintenance period, the median patient age was 41 years and 74% were male. The most common comorbid condition was injection drug use (27%). Overall clinical characteristics were similar between the three periods. The proportion of patients exposed to an antibiotic with broad gram-negative activity was 66% during the pre-intervention period, 33% during the intervention period, and 27% during the maintenance period. The proportion of patients exposed to an antibiotic with antipseudomonal activity was 28%, 18%, and 16% during the pre-intervention, intervention, and maintenance periods, respectively. The median total duration of therapy was 13 (IQR 10-15), 10 (IQR 9-12), and 8 (IQR 7-10) days in the pre-intervention, intervention, and maintenance periods, respectively.

**Figure:**
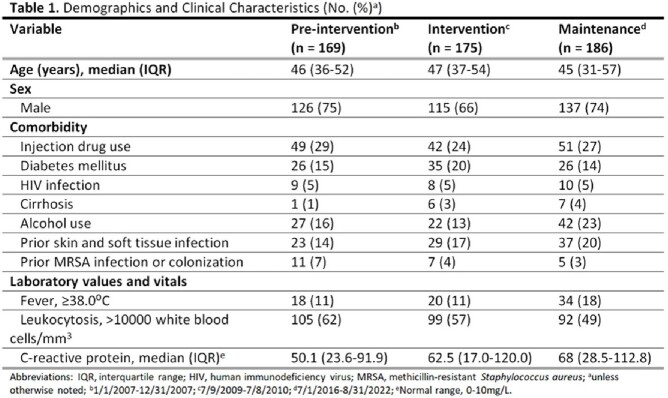

**Figure:**
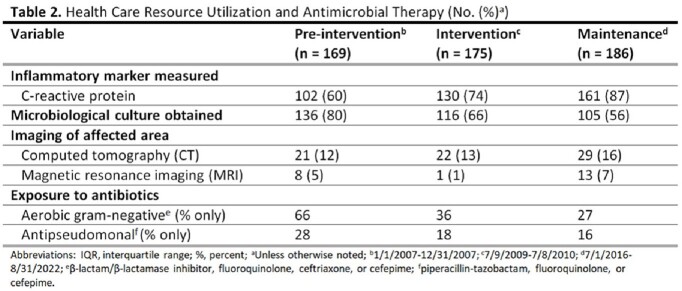

**Figure:**
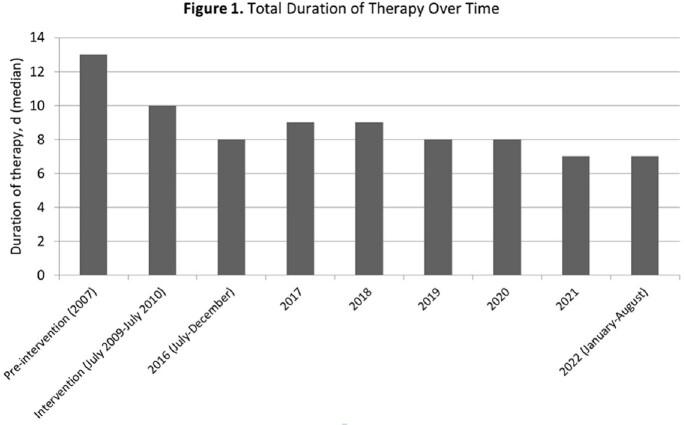

**Conclusion:**

Reductions in use of broad-spectrum antibiotics and durations of therapy for inpatients with SSTIs were sustained 12 years after implementation of a local treatment guideline. These findings add to the evidence that local guidelines are an effective antibiotic stewardship tool and demonstrate that resultant improvements in prescribing can be sustained over prolonged periods.

**Disclosures:**

**Timothy C. Jenkins, MD**, Basilea: Clinical events adjudication committee

